# Content Comparison of Health-Related Quality of Life Measures in Heart Failure Based on the International Classification of Functioning, Disability, and Health: A Systematic Review Protocol 

**Published:** 2018-07

**Authors:** Mahdi Moshki, Haydeh Hashemizadeh, Abdoljavad Khajavi, Shima Minaee, Farveh Vakilian

**Affiliations:** 1 *Social* *Development and Health Promotion Research Center, Gonabad University of Medical Sciences, Gonabad, Iran.*; 2 *Department of Community Medicine, School of Medicine, Gonabad University of Medical Sciences, Gonabad, Iran.*; 3 *Department of Cardiovascular Diseases, Razavi Hospital, Mashhad, Iran.*; 4 *Preventive Atherosclerotic Research Center, Imam Reza Hospital, Faculty of Medicine, Mashhad University of Medical Sciences, Mashhad, Iran* *.*

**Keywords:** *Quality of life*, *Health status*, *Heart failure*, *International classification of functioning, disability and health*

## Abstract

**Background: **Unraveling the relationship between health-related quality of life (HRQOL) instruments and the International Classification of Functioning, Disability, and Health (ICF) seems essential due to the increasing importance of quality of life evaluations in patients with heart failure (HF) and the use of the ICF for comparative purposes. The aim of this study is to identify and compare the content of HRQOL instruments for HF using the ICF coding system.

**Methods** **:** In a 2-stage design, first we will identify all measures used to assess HRQOL for patients with HF and second we will compare the content of those measures using the ICF coding system. Systematic search will be performed in in MEDLINE, CINAHL, and Scopus databases using a combination of free texts and MeSH terms between January 1960 and January 2017. All instruments will be linked to the ICF separately by 2 reviewers according to 10 linking rules developed for this purpose. The degree of agreement between the reviewers will be calculated via the kappa statistic.

**Discussion:** The results of this study may help clinicians and researchers to select the most appropriate outcome measure according to the ICF-based content validity.

## Introduction

Heart failure (HF) constitutes a major public health problem, with a current prevalence of 5.8 million in the USA and 26 million worldwide.^1^^, ^^2^ The incidence of HF in the United States is 870 000 individuals newly diagnosed each year.^3^^, ^^4^ Research in previous years showed that about 2.2% of the population in the United States suffered from HF.^2^^, ^^5^ Since HF is an age-dependent syndrome, its prevalence increases from less than 1% in persons aged under 40 years to more than 10% in those aged over 80 years.^4^^, ^^6^^, ^^7^ Based on the Framingham studies, the incidence of HF is increasing among older individuals, which is concerning given the aging of the population.^8^ Studies foretaste the increase of HF prevalence in the United States up to 46% by the year 2030.^9^ The spreading prevalence of HF might give forth increasing incidence, an aging population, improvements in the treatment of acute cardiovascular disease and HF, or a combination of these factors.^5^

 The primary focus of medical care is the management of chronic diseases, including HF. This program is developed not only to prolong life but also to relieve symptoms and improve overall health.^10^^, ^^11^*According to the World Health Organization* (*WHO)*, being healthy means to have complete state of physical, mental, and social well-being and does not mean not to have any disease or infirmity. Ware (1987) introduced 5 health inherent concepts: physical health, mental health, social functioning, role functioning, and general well-being. He investigated quality of life (QOL) in health sciences through a conservative approach. Because the goal of health care is to maximize the health component of QOL, he suggested the restriction of measures for assessing health status.^12^ QOL is a broad concept covering all aspects of human life whereas**, **health-related quality of life (HRQOL) focuses on the effects of illness and specifically on the impact of treatment on QOL. Regarding health outcomes, most indicators reflect a disease model, whereas HRQOL provides a comprehensive evaluation encompassing all the important aspects of QOL related to health. It has generated a new focus on a broader and more positive concept of health rather than a narrow and negative focus (disease-based).^13^

HRQOL is the main concern of health-care professionals and is becoming an important health outcome indicator.^14^ The measurement of HRQOL in adults with chronic conditions in primary care settings can support patient management and intervention and contribute to service evaluation.^15^

Asking patients how they are or about the effectiveness of treatments is nothing new. HRQOL instruments, however, can provide a formal, standardized, valid, and reliable way of gaining patients’ perspective as to the benefits and limitations of a specific intervention.^16^ The choice of instruments by researchers seems essential in the measurement of HRQOL in patients with HF.^17^

Patient-reported outcomes such as HRQOL measures have proven useful in evaluating the achievement of these goals from the patient’s perspective.^18^^, ^^19^ These outcomes help patients and clinicians make better decisions.^20^ The impact of HF on HRQOL is signiﬁcant^21^^, ^^22^ in comparison with several other common chronic conditions such as hypertension, diabetes, arthritis, chronic lung disease, and angina.^23^^, ^^24^ HRQOL is substantially worse among patients with HF than among individuals without HF and even among patients with other chronic diseases.^25^^, ^^26^

A multidimensional construct, HRQOL is deﬁned as patients’ perceptions of the impact of a medical condition or its treatment on the different aspects of their life including physical functioning, symptom status, psychological status, and social interactions.^27^ Furthermore, as treatment in HF is mainly symptomatic, there has been an increase in interest in assessing HRQOL in patients suffering from HF. Several HF-speciﬁc HRQOL instruments have been developed. Thousands of health status and HRQOL measurement instruments are used in research and clinical practice. Both general and disease-targeted questionnaires are helpful in increasing the understanding of HRQOL outcomes in patients. General instruments, involving health profiles and assessments of the overall health state, compare the relative burden of illness in the general population and between different diseases. Disease-targeted instruments, on the other hand, may be implemented to elucidate the specific domains of particular importance to the patient.^28^

Measures may focus on the symptoms, complaints, disabilities, and disruptions in life that are specific to the clinical condition under study. Indeed, the disease-specific approach has been advocated in the study of arthritis and heart disease as well as the evaluation of chemotherapy.^12^ Clinicians have only recently begun to use these instruments and, therefore, may not yet be completely au fait with the criteria for selecting the most appropriate instrument.^29^

The patient’s perspective is the core of health-care provision and research. From the patient’s perspective, functioning and health are of utmost importance. Generally, any health-care intervention is aimed at restoring impaired body structures and functions, reducing activity limitations and participation restrictions, and preventing the development of new symptoms and disabilities. The increased recognition of the patient’s perspective and, more specifically, functioning and health has brought about an impressive effort in research to develop concepts and instruments to measure them.^30^

Integrating the many competing health status frameworks is possible by linking concepts in one instrument to concepts in another framework using common meaningful terms. Such mappings would augment understanding of the individual concepts upon which the frameworks are built as well as the respective overarching frameworks.^31^ The most comprehensive effort to classify health concepts is the WHO’s International Classification of Functioning, Disability, and Health (ICF).^32^

With the ICF, a universal framework exists in which the items and scales of various HRQOL instruments can be compared in a more desirable way.^33^ The ICF is a multi-purpose classiﬁcation developed to provide a universal language for the explanation of a wide range of health-related phenomena.^34^ The ICF is suggested as a highly useful tool for the comparison of HRQOL instruments. Using the ICF category system as an independent reference to present the contents of measures makes it possible to understand these contents in a comprehensive and standardized manner, thereby facilitating the selection of assessment instruments.^34^^, ^^35^

Due to the increasing importance of HRQOL assessments in patients with HF and due to the increased use of the ICF, it is necessary to find out the relationship between HRQOL instruments and the ICF for comparative purposes. This article is a protocol for a systematic review to appraise the relationship between HRQOL instruments in HF and the ICF. The aims of this study are to identify all the existing disease-speciﬁc questionnaires to measure HRQOL among patients with patients and compare the content of those measures using the ICF coding system.

***Rationale***

The results obtained by the instruments are used by researchers, physicians, and policymakers for further research, evidence-based patient-centered care, guideline development, and evidence-based policymaking. A content comparison is one of the suitable methods that can be applied to select the best available measurement instrument. Moreover, content comparison is a useful tool to see the differences in content between several questionnaires or several performance-based tests. Several reviews deal with the selection of HRQOL instruments for HF, mainly by describing their psychometric properties and other characteristics of the instruments.^20^^, ^^36^ In the past reviews, the validity, practicability, and discrimination of the potential HRQOL instruments were considered and compared. Nevertheless, content comparisons were never represented in the literature as HRQOL instruments for HF. 

***Aims*** 

The objectives of our study are to identify all the existing disease-speciﬁc questionnaires to measure the HRQOL among patients with HF and to compare the content of those measures using the ICF coding system. To that end, we will seek to determine whether the content of HRQOL instruments is represented by the ICF categories and whether the ICF can serve as the common framework when comparing HRQOL instruments. Additionally, areas of the ICF that fail to provide enough detail will be identified in a future revision of the ICF and differences in the contents covered by HRQOL instruments will be examined based on the linkage of their content to the ICF.

## Methods

This is a protocol for a systematic review. The protocol has been registered with PROSPERO (International Prospective Register of Systematic Reviews) (Reference/ID No CRD42015025380). It can be accessed at https://bit.ly/2BhRru2. The review will follow the Preferred Reporting Items for Systematic Reviews and Meta-Analysis (PRISMA-P) flow diagram and guidance set out by the Centre for Reviews and Dissemination.^37^

***Study design***

To approach these 2 objectives, the study will have a 2-stage design. Phase I will identify all measures used to assess HRQOL among patients with HF. We will conduct a systematic literature review to identify disease-specific HRQOL instruments for HF. Phase II will compare the content of those measures using the ICF coding system. We will examine the contents of these instruments by extracting the meaningful concepts contained in the items of the instruments and linking them to the ICF via established linking rules.^30^^, ^^34^ The frequencies of the ICF categories demonstrate the concepts contained in the instruments built on the basis of the descriptive analysis and content comparison.

***Inclusion criteria***

Stage I addresses our first objective. We will include cohort studies, case-control studies, clinical trials, and cross-sectional studies on development study, validation study, reliability, and responsiveness, all of which contribute to HRQOL instruments for HF. These studies will be included because they are the main measurement instruments for the assessment of QOL in patients suffering from HF. Therefore, all articles aimed at reporting or evaluating the measurement properties of the measurement instruments will be included in the present investigation. Linguistic validation studies, all instruments about signs, disease severity measure, disease control measure, and physiology of the heart will be excluded. The study population will consist of patients suffering from HF (chronic HF, severe HF, congestive HF, cardiomyopathy, and left ventricular disease). Participants with other heart diseases like coronary artery disease, myocardial infarction, pectoral angina, atrial fibrillation, peripheral artery disease, stroke, and vascular disease will be excluded. This is a development and validation study. The outcome will contribute to HRQOL, QOL, and health status. The full text of all the instruments should be available. The selection of the studies will be restricted to those published in the English language between January 1960 and January 2017.

***Search strategy***

The aim of this search strategy is to find published studies. In order to identify the existing HF-speciﬁc HRQOL instruments, we will conduct a broad search in MEDLINE (via PubMed), CINAHL (via EBSCO), and Scopus (via Elsevier) databases using a combination of free texts and MeSH terms between January 1960 and January 2017. Filters developed for use with PubMed will be used to search for records relating to patient-reported outcome measures (PROMs). The filters are based on extracts from the search strategy developed for the PROM group bibliography. The Patient-Reported Outcome Measurement Group Department of Public Health FEBRUARY (2010) Citation searches are more sensitive than keyword searches in identifying studies through speciﬁc measurement instruments.^38^ Additionally, citation tracking with references from each article will be performed. Hand searching the reference lists and bibliographies of the included articles, implementation reports, Cochrane Databases of Systematic Reviews (CDSR), Centre for Reviews and Dissemination (CRD), and Cardiovascular Section of the Patient-Reported Outcome and Quality of Life Instruments Database (PROQOLID) (https://eprovide.mapi-trust.org/) will also be considered. Recent systematic reviews that are known to the investigators will be incorporated as well.

***Screening and data extraction***

**Figure 1:**
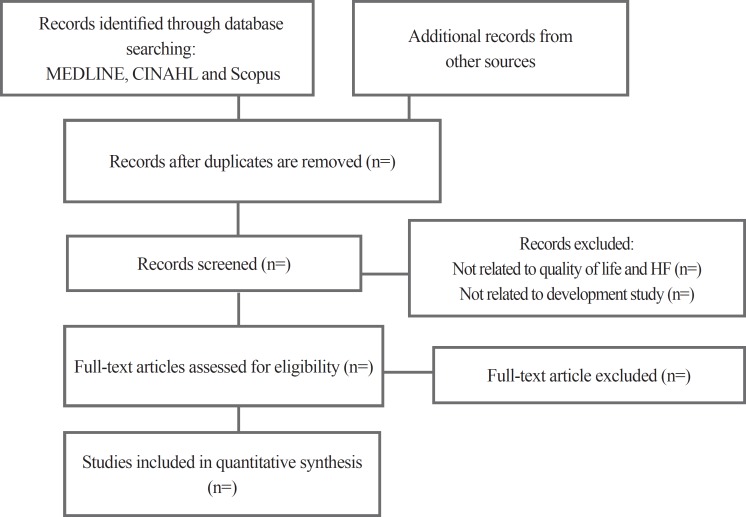
Preferred reporting items for systematic reviews and meta-analyses (PRISMA) flow diagram

**Figure 2:**
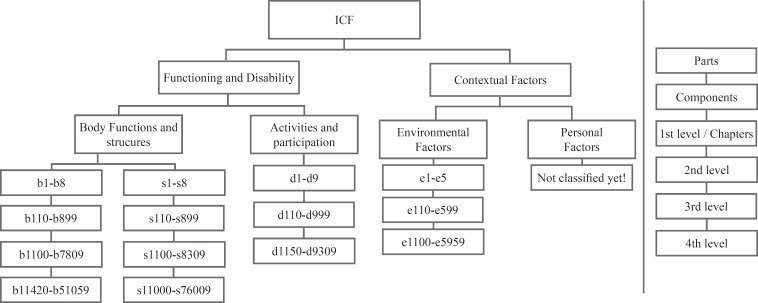
Structure of the International Classification of Functioning, Disability, and Health (ICF) within the chapters (i.e., the ﬁrst item level). Categories can be discriminated (i.e., second- to fourth-item levels).^39^

The retrieved literature will be screened in a 4-stage process. First, all the retrieved literature will be imported into Endnote (Thomson Reuters, Philadelphia, PA, USA) to facilitate the identification and removal of duplicates. Second, 2 reviewers (MM and AK) will independently assess all the retrieved literature for eligibility by title and abstract using the inclusion checklist to ensure accuracy. Any discrepancies in reviewer selections will be resolved through discussion and settled where necessary by a third reviewer (SM). Third, full texts will be obtained for all the remaining citations and reviewed for eligibility by 2 reviewers (MM and AK) and any discrepancies in selection will be resolved through discussion and recourse to a third reviewer (SM), where required. The PRISMA Flow Diagram will be applied to record the number of the studies included and excluded at each stage of the review process (Figure 1). In data extraction, we will proceed through a 2-step procedure. First we will extract the major characteristics of the HRQOL measures such as study title, author, year, journal, study type, data collection method, year of data collection, setting, type of participants, sample size, gender, age groups, nationality, HF class, ejection fraction, and outcome measures. A data extraction form will be used for this phase (Table 1). Next, we will link all the instruments to the ICF. All of the HRQOL instruments in HF will be linked to the ICF separately by 2 reviewers (HH and FV) according to 10 linking rules developed for this purpose. Any discrepancies in the linking process will be resolved through discussion and settled where necessary by a third reviewer (SM). The degree of agreement between the reviewers will be calculated via the kappa statistic. Accordingly, the reliability of the linking process will be assessed using IBM-SPSS-23 and calculating the Kappa coefficient. We will follow the ICF linking guidelines suggested by Cieza et al. which have been practical in a variety of outcome measures in HRQOL measures. The content of the selected measures will be linked to the 2 parts of the ICF. Part I will cover functioning and disability and include body functions (b), body structure (s), and activities and participation (d). Part II will cover contextual factors and include environmental (e) and personal factors (pf). In the classiﬁcation, b, s, d, and e will be followed by a numeric code, starting with the chapter number (1 digit) followed by a second-, third, or fourth-level code (adding 2 digits and 1 digit, respectively). If the provided information by the item is not sufficient to decide which ICF category should be chosen, this item should be labeled nd (not definable). The abbreviation “nd-gh” (not definable-general health) is used for items/concepts concerning health in general and the abbreviation “nd-qol” (not definable-quality of life) is used for items/concepts concerning the QOL of patients in general. If an item is not found in the ICF classification, then this item/concept is labeled “nc” (not covered by the ICF). If an item contains more than 1 concept, each concept will be linked separately.^30^Figure 2 shows the structure of the ICF.^39^

**Table1:** Data extraction form (Phase I)

Study details	
	Study title
	Author
	Year
	Journal
Study characteristics	
	Study type
	Data collection method
	Year of data collection
	Setting
Participant characteristics	
	Type of participants
	Sample size
	Characteristics
	Gender
	Age groups
	Nationality
	Heart failure class
	Ejection fraction
Outcomes	
	Outcome measures

A data extraction tool will be used for the linkage of HRQOL instruments to the respective ICF categories in the component b, s, d, and e (Table 2).^40^ For each of these instruments, we will calculate the reported frequency and the frequency with which its items address b, s, d, and e as well as pf, nc, and nd. If a category is assigned repeatedly in a measure, the category will be counted only once.^41^^, ^^42^ The coding and the linking procedure are depicted in Table 3 as a pilot testing.

Adopted with permission from Elsevier Publication

**Table 2:** Data extraction tool (Phase II)*

Frequency of the ICF components of HF-specific HRQOL instruments
Body functions (b)
b1. Mental function
b2. Sensory functions and pain
b3. Voice and speech functions
b4. Functions of the cardiovascular, hematological, immunological, and respiratory systems
b5. Functions of the digestive, metabolic, and endocrine systems
b6. Genitourinary and reproductive functions
b7. Neuromusculoskeletal and movement-related functions
b8. Functions of the skin and related structures
Body structure (s)
s1. Structures of the nervous system
s2. Eye, ear, and related structures
s3. Structures involved in voice and speech
s4. Structures of the cardiovascular, immunological, and respiratory systems
s5.Structures related to the digestive, metabolic, and endocrine systems
s6. Structures related to the genitourinary and reproductive systems
s7. Structures related to movement
s8. Skin and related structures
Activity and participation (d)
d1. Learning and applying knowledge
d2. General tasks and demands
d3. Communication
d4. Mobility
d5. Self-care
d6. Domestic life
d7. Interpersonal interactions and relationships
d8. Major life areas
d9. Community, social, and civic life
Environmental factors (e)
e1. Products and technology
e2. Natural environment and human-made changes to environment
e3. Support and relationships
e4. Attitudes
e5. Services, systems, and policies

ICF, International Classification of Functioning, Disability, and Health; HF, Heart failure; HRQOL, Health-related quality of life

* Adopted from World Health Organization International Classification of Functioning, Disability and Health (ICF)^40^

**Table 3:** Demonstration of the linking procedure

Item (measure)	Concept	ICF				
		component	Chapter (first level)	Category (second level)	Category (third level)	Category (fourth level)
Because of my heart condition, I suffer from tired legs.	Heart condition	Health condition	Substitute for the underlying disease, classified in the complementary ICD 10			
	I suffer from tired legs.	b	Sensory functions and pain (b2)	Sensation of pain (b280)	Pain in the body part (b2801)	

ICF, International Classification of Functioning, Disability, and Health

***Synthesis***

The synthesis of the results will be presented in 2 stages. First, a descriptive summary including the relevant data that are extracted from eligible studies will be presented, tabulating details about study type, outcome measures, type of participants, sample size, gender, age groups, nationality, HF class, and ejection fraction. Second, a narrative synthesis will be presented and the concepts identified from the instruments will be described according to their frequency distribution across the ICF components. Then, the identified concepts from the instruments will be described according to their frequency distribution across the addressed ICF chapters (first level), categories (second level), third level, and fourth level. Finally, the strengths and limitations of the review will be reported and the implications of the review’s findings for future research, education, policy, and practice will be discussed.

***Validity and rigor***

Six steps have been taken to increase the rigor of the review. First, librarian expert advice was obtained prior to the development of the search strategy and will be applied throughout the systematic review. Second, the filters developed for PubMed will be used to search for records relating to PROMs. Third, validity will be ensured through the use of the PRISMA-P guidelines(41) to ensure the transparent reporting of the review process and findings. Fourth, reliability will be improved through independent assessments of all the retrieved studies for inclusion based on title and abstract, and then full text by 2 reviewers. Fifth, quality assessment will be conducted by 2 reviewers, independently linking all HRQOL instruments to the ICF separately, according to 10 linking rules developed for this purpose and any discrepancies in the linking process will be resolved through discussion and settling where necessary by a third reviewer. The degree of agreement between the reviewers will be calculated by means of the kappa statistic. Sixth, the systematic review is registered with the PROSPERO.

## Discussion

With all the possible instruments available, sometimes it is difficult to opt for the right measurement instrument. The ICF can be a very useful tool because it provides information about the content addressed in the different instruments. According to the linkage, it is possible to assess the heterogeneity of HRQOL instruments in HF regarding the presentation of b, s, d, and e. The comparison of HRQOL instruments in HF may provide nurses, clinicians, and researchers with new insights when selecting HRQOL measures for clinical research.^30^

Moreover, the ICF provides a suitable framework when comparing the content of HRQOL instruments. Content comparison of HRQOL measures in HF confers insights into their differences with respect to the breadth and precision of their coverage of specific concepts.

To our knowledge, this is the first protocol for a systematic review of international evidence concerning the content comparison of HRQOL measures based on the ICF in HF. However, our review has several limitations. First, formulating search terms to only capture HRQOL in HF proved challenging. Nonetheless, we will draw upon filters developed for use with PubMed in order to search for records relating to PROMs. Second, the review will only include studies published in the English language. The inclusion of studies in languages other than English is beyond the scope of the present review because the review team members are not fluent in languages other than English. 

## Notes:

***This paper should be cited as:*** Moshki M, Hashemizadeh H, Khajavi A, Minaee S, Vakilian F. Content Comparison of Health-Related Quality of Life Measures in Heart Failure Based on the International Classification of Functioning, Disability, and Health: A Systematic Review Protocol. J Teh Univ Heart Ctr 2018;13(3):144-152.
